# Cardiometabolic Benefits of a Weight-Loss Mediterranean Diet/Lifestyle Intervention in Patients with Obstructive Sleep Apnea: The “MIMOSA” Randomized Clinical Trial

**DOI:** 10.3390/nu12061570

**Published:** 2020-05-28

**Authors:** Michael Georgoulis, Nikos Yiannakouris, Ioanna Kechribari, Kallirroi Lamprou, Eleni Perraki, Emmanouil Vagiakis, Meropi D Kontogianni

**Affiliations:** 1 Department of Nutrition & Dietetics, School of Health Science & Education, Harokopio University, 70 Eleftheriou Venizelou str., 17671 Athens, Greece; mihalis.georgoulis@gmail.com (M.G.); nyiannak@hua.gr (N.Y.); ikexrimparh@yahoo.gr (I.K.); 2 Center of Sleep Disorders, 1st Department of Critical Care and Pulmonary Services, Medical School of Athens University, Evangelismos Hospital, 45-47 Ipsilantou str., 10676 Athens, Greece; kallirroi76@yahoo.com (K.L.); el1per@msn.com (E.P.); sleeplabathens@yahoo.gr (E.V.)

**Keywords:** obstructive sleep apnea, metabolic syndrome, cardiometabolic profile, weight loss, dietary/lifestyle intervention, Mediterranean diet, Mediterranean lifestyle

## Abstract

Although continuous positive airway pressure (CPAP) is the first-line treatment for obstructive sleep apnea (OSA), its cardiometabolic benefits are questionable. Our aim was to explore whether the combination of a weight-loss Mediterranean diet/lifestyle intervention with OSA standard care leads to greater cardiometabolic improvements compared with standard care alone. We randomly assigned 187 adult, overweight, polysomnography-diagnosed moderate-to-severe OSA patients to a standard care group (SCG, *n* = 65), a Mediterranean diet group (MDG, *n* = 62) or a Mediterranean lifestyle group (MLG, *n* = 60). All three groups were prescribed with CPAP. Additionally, the SCG only received brief written healthy lifestyle advice, while intervention arms were subjected to a six-month weight-loss behavioral intervention based on the Mediterranean diet. The MLG also received guidance for improving physical activity and sleep habits. Glucose metabolism indices, blood lipids, liver enzymes and blood pressure improved only in intervention arms, and were significantly lower compared to the SCG post-intervention (all *p* < 0.05). The age-, sex-, baseline- and CPAP use-adjusted relative risk (95% confidence interval) of metabolic syndrome was 0.58 (0.34–0.99) for the MDG and 0.30 (0.17–0.52) for the MLG compared to the SCG. The MLG additionally presented a lower relative risk of metabolic syndrome compared to the MDG (0.52 (0.30–0.89)). After further adjustment for body-weight change, a lower relative risk of metabolic syndrome was still evident for the MLG compared to the SCG. In conclusion, although standard care alone does not improve OSA patients’ cardiometabolic profile, its combination with a weight-loss Mediterranean diet/lifestyle intervention leads to significant cardiometabolic benefits.

## 1. Introduction

Accumulating evidence suggests that obstructive sleep apnea (OSA) is strongly associated with obesity and cardiometabolic disorders, a fact that has challenged its traditional view as merely an anatomical disease of the upper respiratory system [1]. The relationship between OSA and cardiometabolic health is currently under research, with available data supporting their bidirectional link [2]. On the one hand, obesity is a well-established risk factor for the onset and progression of OSA, given that increased levels of total and abdominal fat have a direct adverse effect on the upper respiratory anatomy, lung volume and breathing control [3]. In addition, the metabolic manifestations of obesity in the context of the metabolic syndrome (MS) contribute to OSA pathogenesis, with central fat accumulation triggering upper airway dysfunction through insulin resistance, inflammation and oxidative stress, and OSA has been proposed to constitute the manifestation of the MS in the respiratory system of overweight and obese individuals [4]. On the other hand, OSA can induce and aggravate obesity and metabolic complications through sleep deprivation and intermittent hypoxia, which induce oxidative stress, inflammation and adipose tissue malfunction, leading to peripheral insulin resistance and ectopic fat deposition [5].

Management of OSA is primarily aiming at preventing sleep-disordered breathing, with continuous positive airway pressure (CPAP) being proposed as the current first-line treatment and offered as standard care in all patients with moderate to severe disease [6]. However, the efficacy of CPAP in improving cardiometabolic risk markers has not been confirmed to date, a fact that significantly limits its value as the sole treatment for the majority of high cardiovascular risk OSA patients [7,8]. Given the close relationship of OSA with obesity, lifestyle-induced weight loss has emerged as a promising complementary treatment for overweight patients [9,10]. However, only few studies have so far explored the efficacy of lifestyle interventions in improving OSA patients’ cardiometabolic profile on top of reducing the disease severity, showing benefits in selected cardiometabolic markers, such as glucose metabolism indices, lipidemic profile, arterial pressure and liver enzymes [11,12,13,14]. In addition, healthy dietary/lifestyle patterns, such as the Mediterranean diet and the Mediterranean lifestyle, could be a valuable therapeutic tool for OSA, targeting not only respiratory lesions but also the cardiometabolic manifestations of the disease [15]; however, evidence regarding their cardiometabolic benefits on OSA patients beyond weight loss is scarce.

The aim of the present study was to explore whether the addition of a weight-loss Mediterranean diet/lifestyle intervention to the current standard care for OSA in clinical practice (i.e., CPAP prescription and brief written healthy lifestyle advice) has an incremental beneficial effect on OSA patients’ cardiometabolic profile, i.e., MS prevalence, glucose metabolism indices, lipidemic profile parameters, liver enzymes and blood pressure, over the effect of standard care alone.

## 2. Materials and Methods

### 2.1. Study Design

The present work is part of the MIMOSA (Mediterranean diet/lifestyle Intervention for the Management of Obstructive Sleep Apnea) study, a 12-month, single-center, single-blind, parallel-group (1:1), randomized, controlled, superiority clinical trial. The MIMOSA study was designed to test the hypothesis that the combination of a weight-loss Mediterranean diet/lifestyle intervention and OSA standard care can yield greater improvements in OSA severity, symptomatology and patients’ cardiometabolic profile compared to standard care alone, and that a Mediterranean lifestyle intervention can lead to additional benefits for OSA patients compared to an intervention based solely on the Mediterranean diet. The MIMOSA study was approved by the Scientific Board of Evangelismos Hospital, Athens, Greece and the Bioethics Committee of Harokopio University, Athens, Greece, was conducted in accordance with the Declaration of Helsinki [16] and is registered in the National Institutes of Health database (ClinicalTrials.gov, NCT02515357).

### 2.2. Participants

Potential candidates for the study were recruited from the Center of Sleep Disorders of “Evangelismos” Hospital. All adult individuals who visited the center and had a clinical suspicion of OSA were subjected to an attended overnight polysomnography (PSG), and those with a definite diagnosis of OSA were referred to the research dietitian for further screening. During screening, anthropometric and blood pressure measurements were taken from all patients and self-reported data were collected on demographic parameters, medical history, medication use, smoking and alcohol drinking. Candidates were considered eligible for participation in the study if they were adult (18–65 years old), overweight or obese (body mass index (BMI) ≥ 25 kg/m^2^) and had moderate or severe OSA (apnea hypopnea index (AHI) ≥ 15 events/h of sleep). Candidates were excluded on the basis of the following: (a) presence of central sleep apnea or other sleep disorders, such as narcolepsy, restless legs syndrome and chronic pain syndrome; (b) presence of chronic diseases, such as diabetes mellitus, cardiovascular disease, familial dyslipidemia, severe uncontrolled hypertension (systolic/diastolic blood pressure > 160/95 mm Hg), chronic kidney disease, malignancy, inflammatory diseases and psychiatric disorders; (c) hospitalization due to acute or chronic respiratory disease or required use of supplemental oxygen during the last year; (d) any kind of surgery during the last three months; (e) pregnancy or breast-feeding for women; (f) use of antipsychotic, antidepressant and other hypnotic drugs, systematic use of steroids or hormone replacement therapy for women; (g) habitual excessive alcohol intake (>210 and >140 g of alcohol per week for men and women, respectively); and (h) currently on a weight-loss diet or recent change in lifestyle habits. Eligible candidates were informed in detail about the aims and procedures of the study and were asked to willingly provide a signed written consent form for participation.

### 2.3. Randomization

Enrolled patients were blindly randomized to one of the three study groups, i.e., a standard care group (SCG) or one of the two intervention arms: a Mediterranean diet group (MDG) and a Mediterranean lifestyle group (MLG). Randomization was based on a restricted minimization approach [17], according to which the first patient was randomly allocated to one of the three study groups using a computerized random number generator and each subsequent patient was sequentially assigned to a particular study group by taking into account previous assignments of participants and specific covariates, namely participants’ age, sex, BMI and AHI levels, in order to minimize baseline between-group imbalances and ensure valid treatment comparisons. The research dietitian was solely responsible for the assignment of participants to interventions, while all other study team members were blinded to patients’ allocation to study groups.

### 2.4. Interventions

After randomization, all three study groups received standard care for OSA management. Specifically, after the initial PSG for OSA diagnosis, all enrolled participants were subjected to an overnight, in-laboratory CPAP titration sleep study according to the American Academy of Sleep Medicine (AASM) guidelines [18] and were accordingly prescribed with the same auto-CPAP device on the basis of current recommendations for OSA management [6]. Patients were given detailed information on CPAP acquisition, use and maintenance and were instructed to use it daily during night sleep for the whole study period. In the context of OSA standard care, patients were also able to monitor CPAP performance and receive reinforcement on optimal use and adherence at bimonthly prescheduled appointments with members of the medical team of the Center of Sleep Disorders. To evaluate compliance with CPAP therapy, patients were asked to record the use of CPAP based on device reports in self-monitoring print forms throughout the six-month intervention period.

On top of CPAP prescription, the SCG received brief written advice for a healthy lifestyle and an indicative hypocaloric daily dietary plan, i.e., 7531 kJ (1800 kcal) for men and 6276 kJ (1500 kcal) for women, without any other intervention until the end of the six-month study period. Patients in the MDG and the MLG participated in an intensive six-month intervention led by the research dietitian that consisted of seven 60-min group (3–5 patients) counselling sessions, held biweekly for the first two months and monthly for the next four months at the laboratory of Clinical Nutrition & Dietetics of Harokopio University. Group sessions were scheduled based on participants’ availability. When an emergency prevented participants from attending a session, they were given the opportunity to reschedule or receive all relevant information through the telephone or an e-meeting with the research dietitian. The intervention was based on cognitive behavioral therapy and several techniques, such as goal setting, problem solving, assessment of readiness and self-efficacy for change, self-monitoring, stimulus control and relapse prevention, were used to facilitate behavior change [19,20,21]. The two principal goals for intervention arms were a 5–10% weight loss and an increase in the level of adherence to the Mediterranean diet, i.e., a dietary pattern characterized by high consumption of olive oil, vegetables, legumes, whole grains, fruits and nuts, moderate consumption of poultry, fish and dairy products, low consumption of red meat products and sweets, and low-to-moderate consumption of wine as the main source of alcohol accompanying main meals (Supplemental Results Table S1) [22]. Briefly, the first session was devoted to weight loss; participants received the same indicative hypocaloric dietary plan as the SCG that served as an example of a balanced diet, and counselling emphasized on dietary practices that can help reduce energy intake, such as the adoption of a balanced daily meal pattern, the avoidance of energy-dense snacks and nibbling, the correct identification of hunger and satiety, the limitation of social overeating and emotional eating, food portion control and the establishment of proper meal conditions (sitting at the table, family or social meals, eating without parallel activities like TV, etc.) In the following six sessions, patients were gradually trained to adopt the Mediterranean diet [22]. In each session, patients were informed about the nutritional value and health effects of specific food groups and were given goals about their recommended consumption frequency and quantity according to the Mediterranean pattern. Other healthy dietary practices, such as ensuring a nutritional variety, choosing unprocessed, traditional, local and seasonal foods, as well as implementing healthy cooking techniques, were also addressed.

Patients in the MLG received the same dietary intervention but were given additional goals for increasing physical activity and achieving an optimal sleep duration and quality. With regard to physical activity, patients were instructed to participate in any kind of aerobic moderate-intensity physical activity (e.g., fast-paced walking, taking the stairs, recreational cycling, recreational swimming, running, dancing or any kind of sports) for ≥150 min/week with emphasis on outdoor and convivial activities than can be maintained in the long term, and to reduce the amount of time spent in sedentary activities (e.g., screen time) to less than 2 h/day, according to the World Health Organization guidelines [23]. To enhance motivation, patients were also given pedometers and were asked to record their total daily steps, aiming at a gradual increase with the ultimate goal of 10,000 steps/day. With regard to sleep habits, patients were instructed to achieve an optimal sleep duration, i.e., 7–9 h/day, as recommended by the AASM [24], and to adopt the traditional Mediterranean mid-day rest (siesta). Education on sleep hygiene was also provided to improve patients’ sleep quality, including guidance on maintaining a steady sleep schedule, ensuring a proper sleep environment, avoiding caffeine, alcohol and foods that can lead to gastrointestinal symptoms before bedtime, as well as avoiding sleeping in the supine position that can aggravate respiratory events.

### 2.5. Assessments

Participants were evaluated in terms of anthropometric parameters, lifestyle habits and cardiometabolic indices both pre- (baseline) and post-intervention (six months).

Body weight and height were measured following standard procedures. Waist circumference (WC) was measured to the nearest 0.1 cm between the lowest rib and the superior border of the iliac crest at the end of normal expiration, using a non-elastic measuring tape positioned parallel to the floor and with the subject standing. Dietary habits were assessed through a validated, 76-item, semi-quantitative food frequency questionnaire [25], based on which dietary intake was expressed in terms of daily food group (e.g., dairy products, fruits, vegetables) and individual food and beverage (e.g., potatoes, olive oil, coffee) consumption. The Mediterranean Diet Score (MedDietScore) [26] was used to evaluate participants’ level of adherence to the Mediterranean diet (range: 0–55; higher values indicate a greater adherence). In addition, two non-consecutive, 24-h dietary recalls were performed both pre- and post-intervention to assess participants’ energy, macronutrient and micronutrient intake. The first recall was performed in a face-to-face interview at the time of the patients’ assessment, while the second one was performed via telephone within seven days. Dietary recalls were obtained using the three-pass approach [27]. To facilitate an accurate portion size evaluation, patients were asked to report quantities of individual foods and beverages consumed in typical household objects (e.g., teaspoons, tablespoons, cups, etc.) and other commonly known items and sizes (e.g., matchbox, cell phone, palm, etc.). For complex food recipes and when common measures were not helpful, a booklet with multiple photos of different portion sizes for several food items was used and patients were asked to indicate their portion size based on the available photos. Each dietary recall was analyzed using Nutritionist ProTM (Axxya Systems, Stafford, TX, USA) [28] and the average of the two recalls was used to report participants’ energy intake. Physical activity level was assessed through the short version of the International Physical Activity Questionnaire [29], based on which, mean daily time (min/day) spent in any kind of physical activity (walking, medium and high intensity activities) was calculated for each participant. Average night-time sleep duration (h/day) was self-reported by participants.

With regard to the assessment of patients’ cardiometabolic profile, fasting blood samples (12 h) were collected post-PSG and plasma aliquots were immediately frozen at −80 °C until analysis. Glucose, total cholesterol (TC), triglycerides (TG) and high-density lipoprotein cholesterol (HDLC) were measured by enzymatic colorimetric assay (COBAS^®^ 8000 analyzer; Roche, Basel, Switzerland). Low-density lipoprotein cholesterol (LDLC) was calculated using the Friedewald formula [30]. Non-HDL cholesterol (nHDLC) (determined as TC minus HDLC), as well as the ratios of TC/HDLC and TG/HDLC, were also calculated. Insulin levels were determined by the chemiluminescence method (E170 modular analyzer; Roche) and the homoeostasis model assessment of insulin resistance (HOMA-IR) was calculated according to Matthews et al. [31]. Liver enzymes, i.e., alanine transferase (ALT), aspartate transferase (AST) and gamma-glutamyl transpeptidase (GGT), were measured by enzymatic colorimetric method (COBAS^®^ 8000 analyzer). Participants’ blood pressures were also measured with an automatic blood pressure monitor operating on the oscillometric principle (OMRON HEM-7130) according to a standardized protocol [32]. All measurements were performed in the left arm, after a 12-h fast and at least a 30-min period without smoking or engaging in any kind of physical activity. After a 5-min rest, the device was used to take two blood pressure measurements separated by a 2-min interval, and their average was used for analyses.

The presence of MS was defined as the coexistence of ≥3 of the five following components, according to the criteria proposed by Alberti et al. [33]: (a) increased WC, i.e., >102 cm for males and >88 cm for females; (b) increased fasting glucose levels, i.e., ≥5.6 mmol/L (≥100 mg/dL) or reception of antidiabetic medication (presence of diabetes mellitus was an exclusion criterion); (c) decreased HDLC levels, i.e., <1.0 mmol/L (<40 mg/dL) for males and <1.3 mmol/L (<50 mg/dL) for females, or reception of relevant medication; (d) increased TG levels ≥1.7 mmol/L (≥150 mg/dL) or reception of lipid-lowering medication; (e) hypertension, i.e., systolic blood pressure (SBP) ≥130 mm Hg or/and diastolic blood pressure (DBP) ≥85 mm Hg, or reception of antihypertensive medication.

### 2.6. Statistical Analysis

The primary endpoint was the prevalence of MS, while secondary endpoints included the prevalence of individual MS components and patients’ cardiometabolic profile indices, i.e., glucose metabolism parameters, lipidemic profile indices, liver enzymes and blood pressure levels. The MIMOSA study was designed and powered to detect post-intervention between-group differences in the AHI as the primary study endpoint. However, the target sample size (*n* = 180) was also sufficient to obtain at least 70% power to detect a significant difference in the prevalence of MS between intervention groups and the SCG at the end of the study allowing for a type-I error rate of 0.05, assuming a 50–70% prevalence of the MS in the total study sample at baseline [34] and a post-intervention decrease of 20–30% in intervention arms on the basis of previous reports [35].

Primary analyses were performed with the intention to treat approach. Missing values were predicted using estimating-equation methods, which involved fitting a statistical model to the observed data with the use of missing-at-random method [36]. A complete-case analysis was also performed in the per protocol population. All analyses were performed using the SPSS version 23.0 (IBM Corp. 2015, Aemonk, NY, USA). The normality of data was assessed through the Shapiro–Wilk test. Results are presented as mean ± standard deviation for normally distributed numerical variables or median (1st, 3rd quartile) for skewed numerical variables, and as absolute number (relative frequency) for categorical variables. Within-group changes were tested through paired samples *t*-test for normally distributed numerical variables or Wilcoxon signed-rank test for skewed numerical variables and McNemar’s test for categorical variables. Between-group differences were tested through analysis of covariance for numerical variables (skewed variables were log transformed and are presented in their anti-logarithm form) and generalized linear models (binomial distribution with logit link function) for categorical variables. The Bonferroni correction [37] was applied to adjust for multiple comparisons. Results are presented as adjusted mean difference and relative risk with their corresponding 95% confidence intervals (CI), respectively. Participants’ age, sex, baseline levels of the dependent variables and CPAP use (h/week) were used as covariates in all basic models, and an additional adjustment was made for percent body-weight change to test the impact of the dietary/lifestyle intervention regardless of the degree of weight loss achieved. A two-sided α level of 5% was used to indicate statistical significance.

## 3. Results

**Figure 1:**
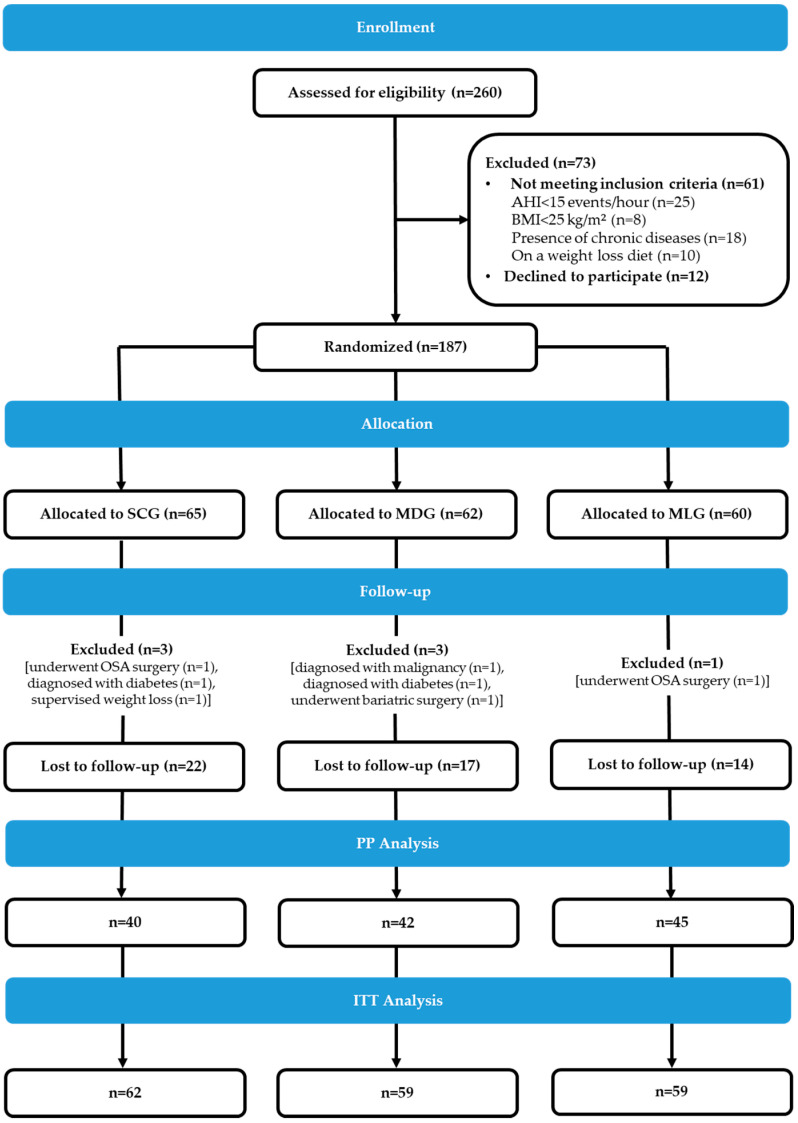
Trial flow diagram. From September 2015 to January 2019, 385 patients were prospectively referred for screening on the basis of their sleep lab files. Of the 385 patients, 60 could not be reached, 50 declined to undergo evaluation and 15 responded positively but did not show up for the scheduled appointment. In total, 260 patients were screened for eligibility. After excluding 61 non-eligible patients and 12 patients who declined participation, 187 provided a signed written consent and were randomized in one of the three study groups. Post-randomization exclusions were limited to seven patients, leaving a total sample of 127 patients for per protocol analysis and 180 patients for intention to treat analysis. The study officially ended in January 2020 when all randomized participants were re-evaluated at 12 months after study initiation. AHI, apnea-hypopnea index; BMI, body mass index; SCG, standard care group; MDG, Mediterranean diet group; MLG, Mediterranean lifestyle group; OSA, obstructive sleep apnea; PP, per protocol; ITT, intention to treat.

The trial flowchart is presented in Figure 1. A total of 260 OSA patients were screened for eligibility, and 187 middle-aged patients (49 ± 10 years) with moderate-to-severe OSA (median (1st, 3rd quartile) AHI: 58 (31–85) events/h; 77% had severe OSA (AHI ≥ 30 events/h)), were eventually recruited and randomized in one of the three study groups, i.e., 65 in the SCG, 62 in the MDG and 60 in the MLG. Baseline characteristics of the total randomized sample and the study groups are presented in Table 1. The majority of patients were men (75%), obese (80%), exhibited a medium adherence to the Mediterranean diet (MedDietScore: 32.1 ± 4.4) and a low physical activity level (median (1st, 3rd quartile) min/d: 13 (2.9, 34)). Regarding their cardiometabolic risk profile, the majority exhibited increased WC values (90%) and hypertension (72%), approximately half (55%) had decreased HDLC, and 43% and 24% had hypertriglyceridemia and hyperglycemia, respectively, with 62% of patients being diagnosed with MS. No significant between-group differences were observed in cardiometabolic or other sociodemographic, anthropometric, lifestyle and clinical characteristics at baseline (Table 1). Although all patients were instructed to initiate CPAP therapy, 82%, 69% and 77% from the SCG, the MDG and the MLG, respectively, actually started the treatment (*p* = 0.2).

A total of seven patients were excluded post-randomization and drop-out rates were 35% (22/62) for the SCG, 29% (17/59) for the MDG and 24% (14/59) for the MLG (Figure 1). Participants in the SCG were considered dropouts if they did not complete the six-month re-evaluation, e.g., could not be reached to schedule the re-evaluation appointment (*n* = 8), refused to participate in the follow-up assessment due to lack of time or interest (*n* = 10) or did not show up for their prescheduled re-evaluation appointment (*n* = 4). Reasons for discontinuing study participation in the intervention arms included circumstances or events that made participation in the counselling sessions not feasible, such as moving out of the city and social/family emergencies (*n* = 14), group-session scheduling conflicts (*n* = 3), the lack of interest in the intervention (*n* = 6), as well as the unjustified absence from ≥2 consecutive counselling sessions and the subsequent complete loss of contact with the research dietitian (*n* = 8). No significant differences were observed between participants who completed the study (*n* = 127) and dropouts (*n* = 53) in terms of several demographic (i.e., age, sex, level of education, financial and employment status), lifestyle (i.e., dietary, physical activity and sleep habits) or clinical characteristics (i.e., body weight status, AHI and OSA severity, presence of the MS) (all *p* ≥ 0.1) (Supplemental Results Table S2).

Participation rate in the counselling sessions was high for both intervention arms, as indicated by the mean number of attended sessions (MDG: 6.38 ± 0.66, MLG: 6.58 ± 0.62, *p* = 0.2). Following the six-month intervention, mean percent body-weight change was −7.4 ± 4.1% for the MDG and −10.6 ± 5.8% for the MLG, both being significantly greater compared to the change of 0.3 ± 3.6% in the SCG (both *p* < 0.001); percent weight loss was also greater for the MLG compared to the MDG (*p* = 0.004). Mean daily energy intake increased in the SCG (+975 kJ/day, *p* < 0.001) but significantly decreased in both intervention arms (MDG: −1134 kJ/day, *p* = 0.005; MLG: −1485 kJ/day, *p* < 0.001). Regarding lifestyle habits, the SCG did not present any improvements in dietary, physical activity or sleep parameters (data not shown). On the contrary, mean MedDietScore increased in both intervention arms (MDG: from 31.8 ± 4.4 to 39.8 ± 4.4; MLG: from 32.3 ± 4.5 to 41.6 ± 3.7, both *p* < 0.001), and at the end of the study its values were significantly higher in both the MDG and the MLG compared to the SCG (both *p* < 0.001). The MLG additionally exhibited an increase in median (1st, 3rd quartile) daily time spent in physical activity (from 17 (6.4, 40) to 50 (34, 60) min/day, *p* < 0.001) and mean sleep duration (from 6.3 ± 1.5 to 7.1 ± 0.6 h/day, *p* < 0.001), with significant differences compared to both the SCG and the MDG at the end of the study (all *p* < 0.001). Among participants who started CPAP treatment (*n* = 135), median (1st, 3rd quartile) use (h/week) was 28 (27, 42) for the SCG, 24 (20, 42) for the MDG and 18 (0.0, 39) for the MLG, with no significant between-group differences (*p* = 0.4). No harms or unintended side-effects from the interventions applied were reported by participants.

Within-group changes in patients’ cardiometabolic profile are presented in Supplemental Results Table S3 and Figure S1. In brief, patients in the SCG experienced a deterioration in their cardiometabolic profile; however, both intervention arms exhibited cardiometabolic improvements, i.e., reductions in insulin, HOMA-IR, TC, nHDLC, TG, TC/HDLC, TG/HDLC, liver enzymes, SBP and DBP, whereas a significant increase in HDLC levels was also observed in the MLG.

Between-group differences in cardiometabolic markers at the end of the study are presented in Table 2. Basic analyses were adjusted for age, sex, baseline values and CPAP use. Compared to the SCG, both the MDG and the MLG exhibited lower values of insulin, HOMA-IR, TG, TC/HDLC, TG/HDLC, ALT, AST, GGT, SBP and DBP, while the MLG additionally presented higher HDLC levels. Comparisons between intervention arms also revealed lower insulin and HOMA-IR, as well as higher HDLC for the MLG compared to the MDG. After further adjustment for body-weight change, both intervention arms still exhibited lower TC/HDLC, TG/HDLC and DBP compared to the SCG, while the MLG also presented lower values of insulin, HOMA-IR and TG, as well as higher values of HDLC compared to the SCG. The only weight-loss adjusted difference between intervention arms was in HDLC, with the MLG presenting higher values compared to the MDG.

With regard to MS prevalence and its individual components (Table 3), the age-, sex-, baseline status- and CPAP use-adjusted relative risk of decreased HDLC was significantly lower in both intervention arms compared to the SCG, the MLG additionally presented a lower relative risk of increased WC, increased TG and hypertension compared to the SCG, while the MDG only showed a trend for a lower risk of increased TG and hypertension compared to the SCG. Moreover, an incremental effect of the Mediterranean lifestyle intervention in reducing the risk of decreased HDLC levels compared to the Mediterranean diet intervention was observed. Regarding the presence of MS, compared to the SCG, both intervention groups showed a lower relative risk after adjustment for age, sex, baseline status and CPAP use. After additional adjustment for percent weight loss, the relative risk (95%CI) of decreased HDLC was 0.40 (0.20, 0.83) for the MDG and 0.18 (0.08, 0.43) for the MLG compared to the SCG, while the MLG still presented a lower relative risk of MS (0.43 (0.20, 0.89)) compared to the SCG, a lower relative risk of decreased HDLC levels compared to the MDG (0.45 (0.25, 0.83)) and a trend for a lower relative risk of MS compared to the MDG.

Analyses in the per protocol population (*n* = 127) confirmed between-group differences in cardiometabolic indices in favor of intervention arms, even though most were attenuated after adjustment for percent weight change (Supplemental Results Table S4). With regard to the risk of MS and its components, differences between the MDG and the SCG were not significant; however, the MLG exhibited a lower relative risk of increased WC compared to the SCG, as well as a lower relative risk of decreased HDLC and MS compared to both the SCG and the MDG; after further adjustment for weight loss, the MLG only exhibited a lower relative risk of decreased HDLC and a trend for a lower relative risk of MS compared to both the SCG and the MDG (Supplemental Results Table S5).

## 4. Discussion

In the present randomized controlled clinical trial among adult, overweight, newly-diagnosed moderate-to-severe OSA patients, the combination of a weight-loss intervention based on the healthy Mediterranean dietary/lifestyle pattern with OSA standard care achieved greater improvements in cardiometabolic parameters, namely insulin resistance, lipidemic profile indices, liver enzymes, blood pressure and the presence of the MS and its components, compared to standard care alone, i.e., prescription of CPAP therapy and brief written lifestyle advice. In addition, the Mediterranean lifestyle intervention, targeting not only a healthy diet but also increased physical activity and optimal sleep habits, combined with standard care, had additional benefits in reducing WC compared to standard care alone, as well as an incremental effect in improving HDLC levels and reducing the risk of MS compared to the Mediterranean diet intervention. All cardiometabolic benefits were evident regardless of CPAP use; however, after adjustment for weight loss, improvements persisted only for the Mediterranean lifestyle intervention.

The relationship between OSA and the MS is well documented [34,38] and efficient strategies for the disease management should target not only sleep-disordered breathing but also its cardiometabolic manifestations. According to meta-analyses of the available, well-designed, randomized controlled clinical trials, CPAP therapy does not seem to improve cardiometabolic indices or cardiovascular morbidity and mortality [8,39,40]. Our results confirm the high prevalence of the MS (approximately 60%) in a sample of adult, overweight, moderate-to-severe OSA patients, and further support that CPAP prescription, traditionally offered alone as standard care to most OSA patients in clinical practice, is unlikely to yield significant cardiometabolic benefits.

The effects of lifestyle weight-loss interventions on OSA management have been thoroughly examined, and the available interventional studies support their efficacy in improving the disease severity, as indicated by significant reductions in the AHI, as well as daytime symptomatology and quality of life, with a clear advantage compared to no intervention [9,10]. Nonetheless, only few studies have explored the potential benefits of weight loss in other endpoints related to cardiometabolic health. In 2014, Chirinos et al. [11] showed that only OSA patients subjected to weight loss, either alone or in combination with CPAP, exhibited a decrease in TG, LDLC and C-reactive protein levels and an increase in insulin sensitivity assessed through a glucose-tolerance test. On the contrary, no improvements were seen in the group of patients receiving CPAP alone, besides a reduction in blood pressure which was evident in all three groups. A few more clinical trials have also reported improvements in individual cardiometabolic indices, such as insulin, lipidemic profile indices (TC, LDLC, HDLC and TG), GGT and blood pressure in patients with OSA after lifestyle-induced weight loss [12,13,14], but no study to date has examined the effect of lifestyle modifications on the presence of the MS. Our study confirms that a weight-loss dietary or lifestyle intervention based on the Mediterranean pattern combined with standard care can produce significant improvements in glucose metabolism parameters, lipidemic profile indices, liver enzymes and blood pressure, and further provides evidence of its superiority in reducing the risk of MS, as compared to standard care alone. The present findings are also in line with current guidelines for the management of overweight and obesity, suggesting that a modest (5–10%) weight loss over six months is sufficient to achieve significant health benefits from decreasing obesity-related comorbidities [41].

In 2011, the Mediterranean lifestyle pyramid was published to replace the older Mediterranean diet pyramid for public health purposes, emphasizing the holistic nature of the healthy way of living of the Mediterranean region that besides prudent dietary choices also includes an adequate physical activity level and optimal sleep habits [22]. In our study, the Mediterranean lifestyle intervention had the most pronounced benefits on OSA patients’ cardiometabolic profile and resulted in a greater increase in HDLC levels and a greater decrease in WC, insulin resistance and the risk of MS compared to the intervention based solely on the Mediterranean diet. Interestingly, the superiority of the Mediterranean lifestyle intervention in increasing HDLC and reducing the risk of MS was evident regardless of percent weight change, suggesting that regular physical activity and optimal sleep habits can have additional benefits for the cardiometabolic health of OSA patients, on top of weight loss achieved through a healthy dietary pattern, and should therefore be part of lifestyle interventions for the management of OSA. A reasonable explanation is the well-established beneficial effect of physical activity on body composition, HDLC levels and insulin sensitivity [42], as well as the beneficial effect of an adequate sleep duration and good quality sleep on glucose metabolism [43].

The MIMOSA study is one of the few available randomized controlled clinical trials that aimed at exploring the effects of a comprehensive behavioral dietary/lifestyle intervention on OSA patients’ cardiometabolic profile. In addition, to our best knowledge, this is the first lifestyle intervention study in which the cardiometabolic profile of OSA patients was assessed not only through individual cardiometabolic indices but also through the cumulative presence of the MS. Although our results point towards the incorporation of dietary/lifestyle interventions in OSA management strategies, our study was a single-center trial and reported findings should be tested in other settings before definite conclusions can be drawn for clinical practice. In addition, standard care in the present study included the prescription but not the provision of a CPAP device, a fact that probably contributed to participants’ low adherence to the treatment. Moreover, although CPAP use was extracted by participants from the device reports, misreporting bias cannot be excluded. Other limitations of the present work include the absence of a group in which no therapy at all was implemented or groups receiving dietary/lifestyle interventions without CPAP or weight loss, due to ethical reasons, as well as the high attrition rate, especially for the SCG, suggesting that these patients were probably looking for a closer monitoring of their diet and lifestyle. Last but not least, we did not systematically record participants’ feedback on the intervention, such as their rating of the content and structure of the intervention, the difficulties and barriers for adhering to intervention goals, as well as their experience with the group nature of counselling sessions and the group leader (research dietitian).

## 5. Conclusions

In conclusion, the addition of a weight-loss Mediterranean diet or lifestyle intervention to OSA standard care, i.e., CPAP prescription and brief written advice on healthy lifestyle, has an incremental effect on improving OSA patients’ cardiometabolic profile over the effect of standard care alone. Interventions incorporating not only healthy dietary patterns, but also adequate physical activity and optimal sleep habits can yield additional weight-loss independent cardiometabolic benefits for OSA patients. Given the limitations of CPAP therapy as the sole treatment for OSA, low-cost behavioral interventions towards modest weight loss and beneficial changes in lifestyle habits that can be implemented in most healthcare settings could constitute a realistic and efficient plan for a comprehensive management of the majority of overweight OSA patients in clinical practice.

## Figures and Tables

**Table 1:** Baseline characteristics of the total randomized sample and the three study groups.

	Total (*n* = 187)	SCG (*n* = 65)	MDG (*n* = 62)	MLG (*n* = 60)	*p* *
Age, years	49 ± 10	48 ± 10	50 ± 9.1	48 ± 10	0.2
Male sex, *n* (%)	141 (75)	51 (79)	44 (71)	46 (77)	0.6
Current smokers, n (%)	61 (33)	19 (29)	26 (42)	16 (27)	0.2
BMI, kg/m^2^	35.6 ± 6.0	36.1 ± 6.4	34.9 ± 5.9	35.8 ± 6.0	0.5
Obesity, *n* (%) ^a^	150 (80)	52 (80)	48 (77)	50 (83)	0.7
Energy intake, kJ/day	9699 ± 3117	9326 ± 3029	10071 ± 3427	9719 ± 2879	0.4
MedDietScore (0–55)	32.1 ± 4.4	32.2 ± 4.5	31.7 ± 4.4	32.4 ± 4.5	0.7
Physical activity, min/day	13 (2.9, 34)	11 (0.0, 34)	11 (0.0, 30)	17 (6.4, 40)	0.2
Sleep duration, h/day	6.1 ± 1.5	6.0 ± 1.8	6.1 ± 1.2	6.2 ± 1.5	0.8
AHI, events/h	58 (31, 85)	52 (31, 87)	60 (35, 81)	62 (21, 89)	0.9
Severe OSA, *n* (%) ^b^	143 (77)	50 (78)	50 (81)	42 (70)	0.4
CPAP therapy, *n* (%)	142 (76)	53 (82)	43 (69)	46 (77)	0.2
Increased WC, *n* (%) ^c^	169 (90)	59 (91)	53 (86)	57 (95)	0.1
Increased TG, *n* (%) ^d^	81 (43)	34 (52)	20 (32)	27 (45)	0.1
Decreased HDLC, *n* (%) ^e^	102 (55)	37 (57)	31 (50)	34 (57)	0.7
Hypertension, *n* (%) ^f^	135 (72)	45 (69)	47 (76)	43 (72)	0.7
Hyperglycemia, *n* (%) ^g^	45 (24)	13 (20)	16 (26)	16 (27)	0.5
Presence of MS, *n* (%) ^h^	115 (62)	42 (65)	31 (50)	42 (70)	0.1

Results are presented as mean ± standard deviation for normally distributed numerical variables or median (1st, 3rd quartile) for skewed numerical variables, and as absolute number (relative frequency) for categorical variables. * *p*-value for the difference between groups, as derived from one-way analysis of variance and Kruskal–Wallis test for the normally distributed and skewed numerical variables, respectively, and chi-square test for categorical variables. Statistical significance level was set at 0.05. ^a^ BMI ≥ 30 kg/m^2^. ^b^ AHI ≥ 30 events/h of sleep. ^c^ >102 cm for males and >88 cm for females. ^d^ ≥1.7 mmol/L (≥150 mg/dL) or reception of lipid-lowering medication. ^e^ <1.0 mmol/L (<40 mg/dL) for males and <1.3 mmol/L (<50 mg/dL) for females, or reception of relevant medication. ^f^ systolic blood pressure ≥130 mm Hg or/and diastolic blood pressure ≥ 85 mm Hg, or reception of antihypertensive medication. ^g^ fasting glucose levels ≥ 5.6 mmol/L (≥100 mg/dL) or reception of antidiabetic medication. ^h^ according to the criteria proposed by Alberti et al. [33]. SCG, standard care group; MDG, Mediterranean diet group; MLG, Mediterranean lifestyle group; BMI, body mass index; MedDietScore, Mediterranean diet score; AHI, apnea-hypopnea index; OSA, obstructive sleep apnea; CPAP, continuous positive airway pressure; WC, waist circumference; TG, triglycerides; HDLC, high-density lipoprotein cholesterol; MS, metabolic syndrome.

**Table 2:** Between-group differences in cardiometabolic markers according to intention to treat analysis (*n* = 180).

	MDG vs. SCG				MLG vs. SCG				MLG vs. MDG			
	MD	95%CI	*p* *	*p* **	MD	95%CI	*p* *	*p* **	MD	95%CI	*p* *	*p* **
Body weight, kg	−8.96	−12.6, −6.33	<0.001	-	−12.6	−15.3, −9.93	<0.001	-	−3.63	−6.29, −0.97	0.004	-
BMI, kg/m^2^	−3.07	−3.93, −2.22	<0.001	-	−4.21	−5.07, −3.35	<0.001	-	−1.14	−2.00, −0.27	0.005	-
WC, cm	−5.51	−8.50, −2.51	<0.001	-	−7.74	−10.8, −4.71	<0.001	-	−2.23	−5.26, 0.80	0.2	-
Glucose, mmol/L ^a^	−0.26	−0.47, −0.05	0.006	0.1	−0.17	−0.38, 0.05	0.2	>0.9	0.10	−0.11, 0.31	0.6	0.4
Insulin, pmol/L ^b^	−24.4	−47.1, −1.64	0.002	0.7	−51.8	−74.8, −28.8	<0.001	0.05	−27.4	−50.3, −4.59	0.05	0.4
HOMA-IR	−1.10	−2.10, −0.10	0.001	0.5	−2.19	−3.21, −1.17	<0.001	0.05	−1.09	−2.11, −0.08	0.02	0.8
TC, mmol/L ^c^	−0.27	−0.57, 0.02	0.08	>0.9	−0.10	−0.40, 0.20	0.5	>0.9	0.17	−0.13, 0.47	>0.9	0.3
LDLC, mmol/L ^c^	−0.15	−0.41, 0.12	0.6	0.8	−0.02	−0.28, 0.25	>0.9	>0.9	0.13	−0.14, 0.40	0.7	0.8
HDLC, mmol/L ^c^	0.05	−0.01, 0.11	0.1	0.08	0.15	0.09, 0.21	<0.001	<0.001	0.09	0.03, 0.15	0.001	<0.001
nHDLC, mmol/L^c^	−0.34	−0.62, −0.06	0.01	0.3	−0.24	−0.53, 0.04	0.1	>0.9	0.10	−0.19, 0.38	>0.9	0.8
TG, mmol/L ^d^	−0.41	−0.68, −0.14	<0.001	0.1	−0.49	−0.76, −0.21	<0.001	0.01	−0.07	−0.35, 0.20	>0.9	>0.9
TC/HDLC	−0.59	−0.93, −0.24	<0.001	0.01	−0.74	−1.09, −0.39	<0.001	0.004	−0.15	−0.50, 0.20	0.9	>0.9
TG/HDLC	−0.52	−0.84, −0.20	<0.001	0.01	−0.72	−1.05, −0.40	<0.001	0.01	−0.20	−0.52, 0.13	0.4	>0.9
ALT, U/L	−2.14	−4.32, −0.04	0.008	0.2	−3.42	−5.64, −1.12	<0.001	0.08	−1.28	−3.50, 0.93	0.5	>0.9
AST, U/L	−4.60	−7.79, −1.41	0.001	0.4	−6.98	−10.2, −3.75	<0.001	0.2	−2.38	−5.62, 0.86	0.5	>0.9
GGT, U/L	−10.1	−17.8, −2.39	<0.001	0.3	−9.66	−17.4, −1.89	<0.001	>0.9	0.43	−7.36, 8.22	>0.9	0.3
SBP, mmHg	−7.29	−12.2, −2.43	0.001	0.5	−6.93	−11.8, −2.04	0.002	>0.9	0.37	−4.49, 5.22	>0.9	>0.9
DBP, mmHg	−9.52	−13.4, −5.63	<0.001	0.001	−6.70	−10.6, −2.81	<0.001	0.01	2.82	−1.08, 6.71	0.3	0.6

Results are presented as mean difference (95% confidence interval). * *p*-value for the difference between groups at the end of the study, as derived from analysis of covariance (skewed variables were log-transformed and are presented in their anti-logarithm form; Bonferroni correction was applied to adjust for multiple comparisons). All models were adjusted for participants’ age, sex, baseline levels of the dependent variables and continuous positive away pressure use (h/week). Statistical significance level was set at 0.05. ** *p*-value after further adjustment for body-weight change (%). ^a^ to convert glucose to mg/dL multiply mmol/L by 18.018. ^b^ to covert insulin to μIU/L multiply pmol/L with 0.167. ^c^ to convert cholesterol to mg/dL multiply mmol/L by 38.610. ^d^ to convert triglycerides to mg/dL multiply mmol/L by 88.496. MDG, Mediterranean diet group; SCG, standard care group; MLG, Mediterranean lifestyle group; MD, mean difference; CI, confidence interval; BMI, body mass index; WC, waist circumference; HOMA-IR, homeostasis model of assessment of insulin resistance; TC, total cholesterol; LDLC, low-density lipoprotein cholesterol; HDLC, high-density lipoprotein cholesterol; TG, triglycerides; ALT, alanine transferase; AST, aspartate transferase; GGT, gamma-glutamyl transpeptidase; SBP, systolic blood pressure; DBP, diastolic blood pressure.

**Table 3:** Between-group differences in the presence of the metabolic syndrome and its components according to intention to treat analysis (*n* = 180).

	MDG vs. SCG				MLG vs. SCG				MLG vs. MDG			
	RR	95%CI	*p* *	*p* **	RR	95%CI	*p* *	*p* **	RR	95%CI	*p* *	*p* **
Increased WC ^a^	0.68	0.17, 2.70	0.6	0.9	0.22	0.06, 0.85	0.02	0.2	0.33	0.12, 0.91	0.04	0.2
Increased TG ^b^	0.51	0.26, 0.99	0.08	>0.9	0.46	0.24, 0.90	0.05	>0.9	0.92	0.45, 1.85	0.8	>0.9
Decreased HDLC ^c^	0.54	0.30, 0.98	0.04	0.01	0.27	0.14, 0.52	<0.001	<0.001	0.51	0.29, 0.90	0.03	0.01
Hypertension ^d^	0.60	0.35, 0.99	0.09	>0.9	0.38	0.23, 0.64	<0.001	>0.9	0.64	0.39, 1.06	0.09	>0.9
Hyperglycemia ^e^	0.68	0.39, 1.19	0.3	0.7	0.60	0.34, 1.07	0.2	0.7	0.88	0.49, 1.59	0.7	0.7
Presence of MS ^f^	0.58	0.34, 0.99	0.04	0.4	0.30	0.17, 0.52	<0.001	0.05	0.52	0.30, 0.89	0.04	0.08

Results are presented as relative risk (95% confidence interval). * *p*-value for the difference between groups at the end of the study, as derived from generalized linear models (binomial distribution with logit link function; Bonferroni correction was applied to adjust for multiple comparisons). All models were adjusted for participants’ age, sex, baseline levels of the dependent variables and continuous positive airway pressure use (h/week). Statistical significance level was set at 0.05. ** *p*-value after further adjustment for body-weight change (%). ^a^ >102 cm for males and >88 cm for females. ^b^ ≥1.7 mmol/L (≥150 mg/dL) or reception of lipid-lowering medication. ^c^ <1.0 mmol/L (<40 mg/dL) for males and <1.3 mmol/L (<50 mg/dL) for females, or reception of relevant medication. ^d^ systolic blood pressure ≥130 mm Hg or/and diastolic blood pressure ≥ 85 mm Hg, or reception of antihypertensive medication. ^e^ fasting glucose levels ≥ 5.6 mmol/L (≥100 mg/dL) or reception of antidiabetic medication. ^f^ according to the criteria proposed by Alberti et al. [33]. MDG, Mediterranean diet group; SCG, standard care group; MLG, Mediterranean lifestyle group; RR, relative risk; CI, confidence interval; WC, waist circumference; TG, triglycerides; HDLC, high-density lipoprotein cholesterol; MS, metabolic syndrome.

## Supplementary Materials

The following are available online at https://www.mdpi.com/2072-6643/12/6/1570/s1, Figure S1: Within-group changes in the presence of the metabolic syndrome and its components according to intention to treat analysis (*n* = 180), Table S1: Content and goals of the dietary intervention, Table S2: Baseline characteristics of participants who completed and those who dropped out of the study, Table S3: Within-group changes in cardiometabolic markers according to intention to treat analysis (*n* = 180), Table S4: Between-group differences in cardiometabolic markers according to per protocol analysis (*n* = 127), Table S5: Between-group differences in the presence of the metabolic syndrome and its components according to per protocol analysis (*n* = 127).
