# Supplementary material for: Cardiometabolic Benefits of a Weight-Loss Mediterranean Diet/Lifestyle Intervention in Patients with Obstructive Sleep Apnea: The “MIMOSA” Randomized Clinical Trial
Source: Nutrients. 2020 May 28;12(6):1570. doi: 10.3390/nu12061570 (PMC7352432; doi:10.3390/nu12061570)
Supplement: Supplementary file 1 [file nutrients-12-01570-s001.zip › nutrients-798364-supplementary.docx]

**SUPPLEMENTAL RESULTS**

Cardiometabolic benefits of a weight-loss Mediterranean diet/lifestyle intervention in patients with obstructive sleep apnea: the "MIMOSA" randomized clinical trial.

Michael Georgoulis ^1^, Nikos Yiannakouris ^1^, Ioanna Kechribari ^1^, Kallirroi Lamprou ^2^, Eleni Perraki ^2^, Emmanouil Vagiakis ^2^ and Meropi D Kontogianni ^1,^*

^1^ Department of Nutrition & Dietetics, School of Health Science & Education, Harokopio University, 70 Eleftheriou Venizelou str., 17671 Athens, Greece; [mihalis.georgoulis@gmail.com](mailto:mihalis.georgoulis@gmail.com) (M.G.); [nyiannak@hua.gr](mailto:nyiannak@hua.gr) (N.Y.); [ikexrimparh@yahoo.gr](mailto:ikexrimparh@yahoo.gr) (Ι.Κ.); [mkont@hua.gr](mailto:mkont@hua.gr) (M.D.K.)

^2^ Center of Sleep Disorders, 1^st^ Department of Critical Care and Pulmonary Services, Medical School of Athens University, Evangelismos Hospital, 45-47 Ipsilantou str., 10676 Athens, Greece; [kallirroi76@yahoo.com](mailto:kallirroi76@yahoo.com) (K.L.); [el1per@msn.com](mailto:el1per@msn.com) (E.P.); [sleeplabathens@yahoo.gr](mailto:sleeplabathens@yahoo.gr) (E.V.)

***** Correspondence: [mkont@hua.gr](mailto:mkont@hua.gr); Tel: +30 210 9549359, Fax: +30 210 9549141

Table S1. Content and goals of the dietary intervention.

| **Session 1: Weight loss and healthy dietary practices.**  Individualized weight loss goal of 5-10% through a negative energy balance. Frequent and nutritionally complete meals (3 main meals plus healthy snacks if necessary), correct identification of hunger and satiety, portion control and proper meal conditions. |
| --- |
| **Session 2: Fruits and vegetables.**  Consumption of 3-6 servings of fruits and ≥2 servings of vegetables per day. Emphasis was given on selection of fruits and vegetables based on variety, locality and seasonality. |
| **Session 3: Grains and dairy products.**  Consumption of 3-6 servings of grains and 2-3 servings of dairy products per day. Emphasis was given on the substitution of refined with whole grains. |
| **Session 4: Fish-seafood and white meat.**  Consumption of ≥2 servings of fish - seafood and 2 servings of white meat per week. Emphasis was given on the selection of small and fatty fish and lean white meat. |
| **Session 5: Legumes and olive oil.**  Consumption of ≥3 portions of legumes per week. Use of olive oil as the main source of fat and preference for extra virgin olive oil. Education on different types of fat, their dietary sources and their health effects, as well as on the limitation of trans fat intake. |
| **Session 6: Red meat & processed meat.**  Consumption of ≤1 serving of red meat and ≤1 serving of processed meat products per week. Emphasis was given on the selection of lean red meat, e.g. sirloin and round, as well as the removal of visible fat before consumption. |
| **Session 7: Sugar, salt and alcohol.**  Reduction in sugar and salt intake, and prudent alcohol consumption (≤1 portion and ≤2 portions of alcohol per day for women and men, respectively, if alcohol is consumed). |

Table S2**. Baseline characteristics of participants who completed and those who dropped out of the study.**

|  | **Completers (n=127)** | **Dropouts (n=53)** | **P*** |
| --- | --- | --- | --- |
| Age, y | 49 ± 11 | 48 ± 8.1 | 0.4 |
| Male sex, n (%) | 92 (72) | 43 (81) | 0.1 |
| Education, y | 14.1 ± 3.4 | 14.1 ± 3.6 | >0.9 |
| Annual income level ^a^ |  |  | 0.6 |
| low | 53 (42) | 19 (36) |  |
| medium | 54 (42) | 25 (47) |  |
| high | 20 (16) | 9 (17) |  |
| Employment status, n (%) ^b^ |  |  | 0.2 |
| state employees | 24 (19) | 13 (25) |  |
| private employees | 41 (32) | 23 (43) |  |
| self-employed | 27 (21) | 10 (19) |  |
| not employed | 35 (28) | 7 (13) |  |
| Current smokers, n (%) | 40 (31) | 18 (34) | 0.6 |
| BMI, kg/m^2^ | 35.8 ± 5.7 | 35.2 ± 7.0 | 0.6 |
| Obesity, n (%) ^c^ | 105 (83) | 42 (79) | 0.4 |
| MedDietScore (0-55) | 32.2 ± 4.3 | 31.9 ± 4.8 | 0.9 |
| Physical activity, min/d | 14.3 (4.3, 33) | 10.7 (0.0, 43) | 0.6 |
| Sleep duration, h/d | 6.2 ± 1.4 | 6.0 ± 1.8 | 0.5 |
| AHI, events/h | 59 (33-87) | 53 (26-74) | 0.6 |
| Severe OSA, n (%) ^d^ | 99 (78) | 39 (74) | 0.4 |
| CPAP therapy, n (%) | 98 (77) | 40 (75) | 0.6 |
| Presence of MS, n (%) ^e^ | 75 (59) | 35 (66) | 0.4 |

Results are presented as mean ± standard deviation for normally distributed numerical variables or median (1^st^, 3^rd^ quartile) for skewed numerical variables, and as absolute number (relative frequency) for categorical variables.

* P-value for the difference between patients who completed and those who dropped out of the study, as derived from Student’s t-test and Man-Whitney U test for normally distributed and skewed numerical variables, respectively, and chi-square test for categorical variables. Statistical significance level was set at 0.05.

^a^ low: ≤10.000 euros/y; medium: 10.000-20.000 euros/y; high: >20.000 euros/y. ^b^ Unemployed and retired individuals, as well as housekeepers and students were considered as not employed. ^c^ BMI≥30 kg/m^2^. ^d^ AHI≥30 events/h of sleep. ^e^ According to the criteria proposed by Alberti et al.^[[1]](#footnote-1)^.

BMI, body mass index; MedDietScore, Mediterranean diet score; AHI, apnea-hypopnea index; OSA, obstructive sleep apnea; CPAP, continuous positive airway pressure; MS, metabolic syndrome.

**Table S3. Within-group changes in cardiometabolic markers according to intention to treat analysis (n=180).**

|  | **SCG (n=62)** | | | **MDG (n=59)** | | | **MLG (n=59)** | | |
| --- | --- | --- | --- | --- | --- | --- | --- | --- | --- |
|  | **Baseline** | **6 months** | **P*** | **Baseline** | **6 months** | **P*** | **Baseline** | **6 months** | **P*** |
| Body weight, kg | 111 ± 22 | 111 ± 20 | >0.9 | 108 ± 24 | 99 ± 22 | <0.001 | 108 ± 20 | 96 ± 16 | <0.001 |
| BMI, kg/m^2^ | 35.8 ± 6.3 | 35.8 ± 5.9 | >0.9 | 34.8 ± 5.9 | 32.2 ± 5.4 | <0.001 | 35.5 ± 5.6 | 31.6 ± 4.4 | <0.001 |
| WC, cm | 118 ± 14 | 118 ± 15 | 0.8 | 118 ± 17 | 112 ± 15 | <0.001 | 117 ± 14 | 110 ± 10 | <0.001 |
| Glucose, mmol/L ^a^ | 4.99 (4.83, 5.38) | 5.22 (4.94, 5.55) | 0.001 | 5.11 (4.83, 5.55) | 5.00 (4.66, 5.38) | 0.1 | 5.11 (4.88, 5.55) | 5.13 (4.98, 5.44) | 0.9 |
| Insulin, pmol/L ^b^ | 80 (56, 143) | 94 (73, 156) | <0.001 | 82 (52, 124) | 68 (49, 98) | 0.004 | 100 (69, 141) | 67 (47, 84) | <0.001 |
| HOMA-IR | 2.93 (2.05, 4.99) | 3.70 (2.62, 6.22) | <0.001 | 3.35 (1.84, 4.76) | 2.61 (1.74, 3.89) | <0.001 | 3.88 (2.38, 5.24) | 2.48 (1.76, 3.34) | <0.001 |
| TC, mmol/L ^c^ | 4.88 ± 0.95 | 4.86 ± 0.78 | 0.8 | 4.88 ± 0.97 | 4.56± 0.91 | <0.001 | 4.78 ± 0.86 | 4.60 ± 0.92 | 0.03 |
| LDLC, mmol/L ^c^ | 3.08 ± 0.85 | 3.08 ± 0.69 | 0.9 | 3.09 ± 0.74 | 2.91 ± 0.75 | 0.001 | 2.93 ± 0.81 | 2.88 ± 0.85 | 0.6 |
| HDLC, mmol/L ^c^ | 1.06 ± 0.24 | 0.98 ± 0.22 | <0.001 | 1.11 ± 0.27 | 1.08 ± 0.26 | 0.9 | 1.05 ± 0.24 | 1.11 ± 0.24 | <0.001 |
| nHDLC, mmol/L ^c^ | 3.82 ± 0.97 | 3.88 ± 0.82 | 0.5 | 3.76 ± 0.98 | 3.47 ± 0.89 | <0.001 | 3.73 ± 0.87 | 3.48 ± 0.92 | 0.002 |
| TG, mmol/L ^d^ | 1.55 (1.07, 1.87) | 1.59 (1.09, 1.98) | 0.007 | 1.16 (0.92, 1.65) | 1.07 (0.82, 1.49) | <0.001 | 1.41 (1.21, 2.37) | 1.33 (0.95, 1.56) | <0.001 |
| TC/HDLC | 4.80 ± 1.37 | 5.18 ± 1.34 | <0.001 | 4.60 ± 1.31 | 4.39 ± 1.23 | 0.03 | 4.83 ± 1.58 | 4.30 ± 1.32 | <0.001 |
| TG/HDLC | 1.50 (0.92, 2.19) | 1.60 (1.09, 2.24) | 0.001 | 1.16 (0.82, 1.76) | 1.06 (0.78, 1.43) | 0.009 | 1.41 (0.96, 2.42) | 1.12 (0.79, 1.64) | <0.001 |
| ALT, U/L | 20 (17, 23) | 21 (18, 23) | 0.07 | 19 (16, 23) | 19 (16, 22) | 0.08 | 21 (17, 26) | 19 (16, 22) | <0.001 |
| AST, U/L | 24 (17, 35) | 21 (17, 32) | 0.02 | 21 (16, 27) | 17 (13, 19) | <0.001 | 26 (20, 34) | 17 (15, 22) | <0.001 |
| GGT, U/L | 27 (19, 39) | 30 (20, 40) | <0.001 | 21 (18, 32) | 19 (15, 26) | <0.001 | 25 (17, 42) | 19 (13, 36) | <0.001 |
| SBP, mmHg | 128 ± 12 | 129 ± 14 | 0.4 | 136 ± 18 | 124 ± 9 | <0.001 | 132 ± 14 | 122 ± 9 | <0.001 |
| DBP, mmHg | 83 ± 10 | 87 ± 10 | 0.007 | 88 ± 15 | 79 ± 8 | <0.001 | 86 ± 13 | 81 ± 7 | 0.002 |

Results are presented as mean ± standard deviation for normally distributed numerical variables or median (1^st^, 3^rd^ quartile) for skewed numerical variables.

* P-value for the within-group change, as derived from paired samples t-test and Wilcoxon signed-rank test for normally distributed and skewed numerical variables, respectively. Statistical significance level was set at 0.05.

^a^ to convert glucose to mg/dL multiply mmol/L by 18.018. ^b^ to covert insulin to μIU/L multiply pmol/L with 0.167. ^c^ to convert cholesterol to mg/dL multiply mmol/L by 38.610. ^d^ to convert triglycerides to mg/dL multiply mmol/L by 88.496.

SCG, standard care group; MDG, Mediterranean diet group; MLG, Mediterranean lifestyle group; BMI, body mass index; WC, waist circumference; HOMA-IR, homeostasis model of assessment of insulin resistance; TC, total cholesterol; LDLC, low-density lipoprotein cholesterol; HDLC, high-density lipoprotein cholesterol; TG, triglycerides; ALT, alanine transferase; AST, aspartate transferase; GGT, gamma-glutamyl transpeptidase; SBP, systolic blood pressure; DBP, diastolic blood pressure.


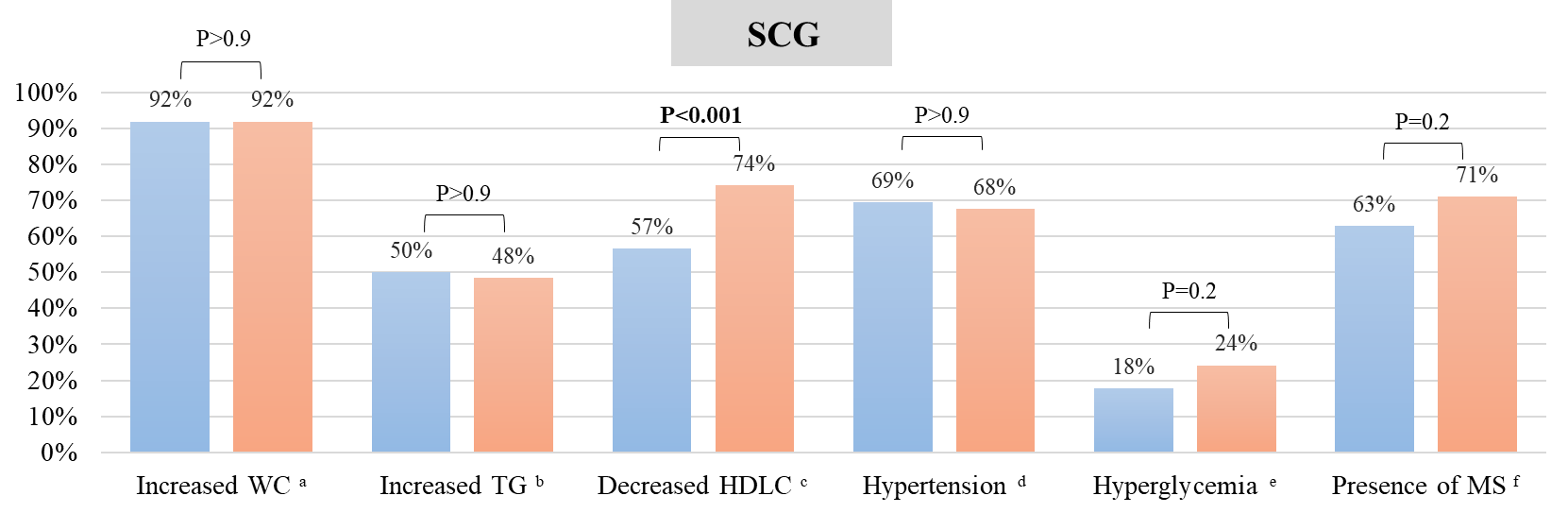


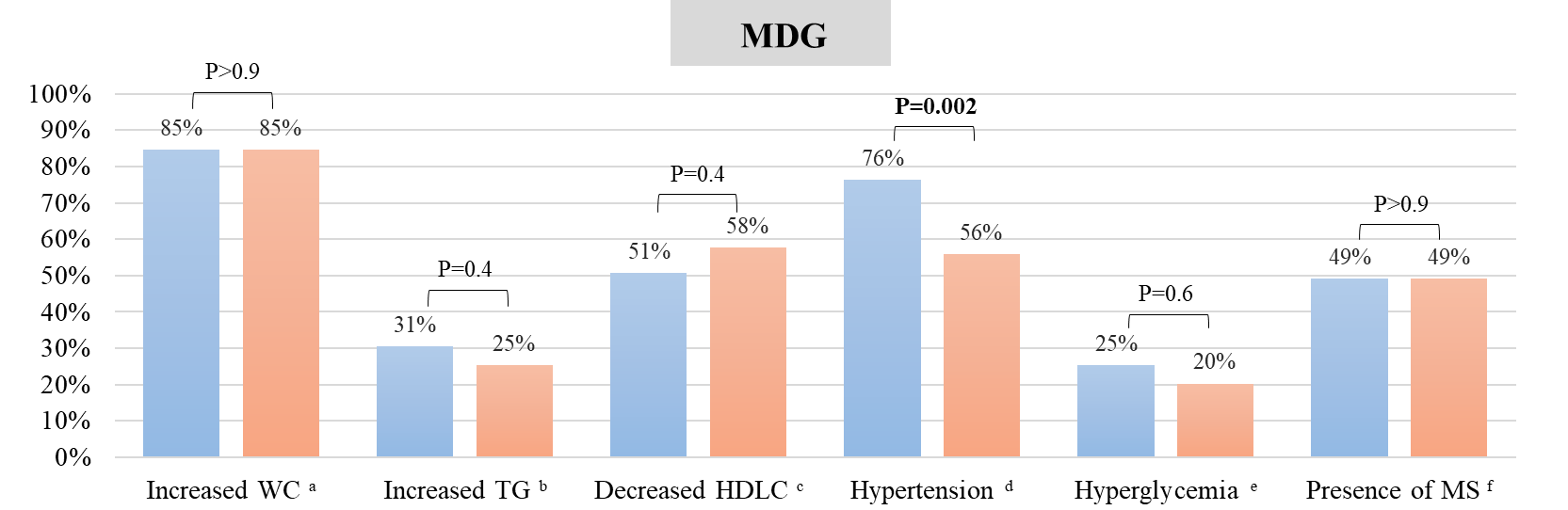


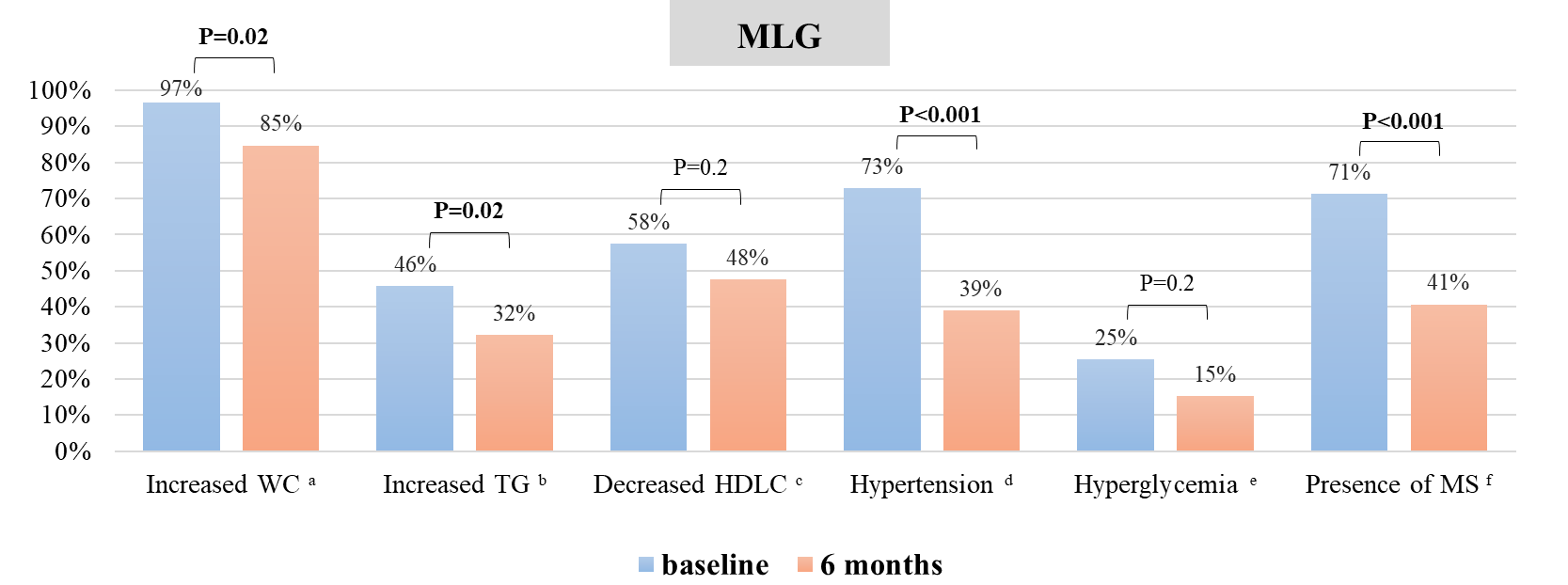


**Figure S1. Within-group changes in the presence of the metabolic syndrome and its components according to intention to treat analysis (n=180).**

Results are presented as % frequency. Within group changes were tested through McNemar's test. Statistical significance level was set at 0.05.

^a^ >102 cm for males and >88 cm for females. ^b^ ≥1.7 mmol/L (≥150 mg/dL) or reception of lipid-lowering medication. ^c^ <1.0 mmol/L (<40 mg/dL) for males and <1.3 mmol/L (<50 mg/dL) for females, or reception of relevant medication. ^d^ systolic blood pressure ≥130 mm Hg or/and diastolic blood pressure ≥85 mm Hg, or reception of antihypertensive medication. ^e^ fasting glucose levels ≥5.6 mmol/L (≥100 mg/dL) or reception of antidiabetic medication. ^f^ According to the criteria proposed by Alberti et al.^[[2]](#footnote-2)^.

SCG, standard care group; WC, waist circumference; TG, triglycerides; HDLC, high-density lipoprotein cholesterol; MS, metabolic syndrome; MDG, Mediterranean diet group; MLG, Mediterranean lifestyle group.

**Table S4. Between-group differences in cardiometabolic markers according to per protocol analysis (n=127).**

|  | **MDG vs. SCG** | | | | **MLG vs. SCG** | | | | **MLG vs. MDG** | | | |
| --- | --- | --- | --- | --- | --- | --- | --- | --- | --- | --- | --- | --- |
|  | **MD** | **95%CI** | **P*** | **P**** | **MD** | **95%CI** | **P*** | **P**** | **MD** | **95%CI** | **P*** | **P**** |
| Body weight, kg | -8.74 | -12.0, -5.46 | <0.001 | - | -12.4 | -15.6, -9.13 | <0.001 | - | -3.63 | -6.83, -0.43 | 0.02 | - |
| BMI, kg/m^2^ | -2.86 | -3.92, -1.79 | <0.001 | - | -3.91 | -4.95, -2.86 | <0.001 | - | -1.05 | -2.08, -0.02 | 0.04 | - |
| WC, cm | -6.19 | -9.86, -2.52 | <0.001 | - | -8.52 | -12.1, -4.94 | <0.001 | - | -2.33 | -5.89, 1.22 | 0.3 | - |
| Glucose, mmol/L ^a^ | -0.28 | -0.59, 0.04 | 0.1 | 0.3 | -0.14 | -0.47, 0.18 | 0.9 | >0.9 | 0.13 | -0.15, 0.43 | 0.5 | 0.4 |
| Insulin, pmol/L ^b^ | -25.5 | -56.9, 5.88 | 0.2 | >0.9 | -51.0 | -82.8, -18.9 | 0.001 | 0.4 | -25.5 | -53.5, 2.46 | 0.3 | 0.9 |
| HOMA-IR | -1.17 | -2.56, -0.21 | 0.02 | 0.8 | -2.18 | -3.59, -0.77 | 0.001 | 0.4 | -1.01 | -2.24, 0.23 | 0.7 | >0.9 |
| TC, mmol/L ^c^ | -0.29 | -0.73, 0.15 | 0.3 | >0.9 | -0.09 | -0.54, 0.37 | >0.9 | >0.9 | 0.20 | -0.19, 0.60 | 0.6 | 0.4 |
| LDLC, mmol/L ^c^ | -0.15 | -0.54, 0.24 | >0.9 | >0.9 | 0.01 | -0.39, 0.40 | >0.9 | >0.9 | 0.16 | -0.19, 0.50 | 0.8 | 0.8 |
| HDLC, mmol/L ^c^ | 0.05 | -0.04, 0.14 | 0.6 | 0.4 | 0.15 | 0.05, 0.24 | 0.001 | 0.001 | 0.10 | 0.02, 0.18 | 0.01 | 0.009 |
| nHDLC, mmol/L ^c^ | -0.35 | -0.77, 0.08 | 0.2 | 0.7 | -0.23 | -0.66, 0.21 | 0.6 | >0.9 | 0.12 | -0.26, 0.49 | >0.9 | >0.9 |
| TG, mmol/L ^d^ | -0.43 | -0.82, -0.04 | 0.001 | 0.2 | -0.50 | -0.90, -0.09 | 0.001 | 0.8 | -0.07 | -0.42, 0.28 | >0.9 | >0.9 |
| TC/HDLC | -0.54 | -1.06, -0.03 | 0.03 | 0.2 | -0.70 | -1.23, -0.17 | 0.005 | 0.1 | -0.16 | -0.61, 0.30 | >0.9 | >0.9 |
| TG/HDLC | -0.51 | -0.96, -0.06 | 0.001 | 0.06 | -0.70 | -1.16, -0.24 | <0.001 | 0.05 | -0.19 | -1.36, 0.48 | >0.9 | >0.9 |
| ALT, U/L | -1.83 | -5.13, 1.46 | 0.09 | 0.5 | -3.28 | -6.68, -0.19 | 0.02 | 0.3 | -1.45 | -0.59, 0.21 | >0.9 | >0.9 |
| AST, U/L | -4.03 | -8.81, 0.74 | 0.07 | >0.9 | -6.45 | -11.4, -1.54 | 0.004 | 0.8 | -2.42 | -6.68, 1.84 | 0.7 | >0.9 |
| GGT, U/L | -8.54 | -20.2, -3.08 | 0.008 | >0.9 | -8.56 | -20.5, -3.34 | 0.01 | >0.9 | -0.02 | -10.4, 10.3 | >0.9 | 0.9 |
| SBP, mmHg | -8.54 | -15.4, -1.71 | 0.009 | 0.6 | -8.39 | -15.4, -1.36 | 0.01 | >0.9 | 0.15 | -6.15, 6.45 | >0.9 | >0.9 |
| DBP, mmHg | -10.6 | -16.1, -5.17 | <0.001 | 0.007 | -8.20 | -13.8, 2.61 | 0.002 | 0.4 | 2.42 | -2.61, 7.45 | 0.7 | 0.3 |

Results are presented as mean difference (95% confidence interval).

* P-value for the difference between groups at the end of the study, as derived from analysis of covariance (skewed variables were log-transformed and are presented in their anti-logarithm form; Bonferroni correction was applied to adjust for multiple comparisons). All models were adjusted for participants’ age, sex, baseline levels of the dependent variables and continuous positive away pressure use (h/week). Statistical significance level was set at 0.05. ** P-value after further adjustment for body-weight change (%).

^a^ to convert glucose to mg/dL multiply mmol/L by 18.018. ^b^ to covert insulin to μIU/L multiply pmol/L with 0.167. ^c^ to convert cholesterol to mg/dL multiply mmol/L by 38.610. ^d^ to convert triglycerides to mg/dL multiply mmol/L by 88.496.

MDG, Mediterranean diet group; SCG, standard care group; MLG, Mediterranean lifestyle group; MD, mean difference; CI, confidence interval; BMI, body mass index; WC, waist circumference; HOMA-IR, homeostasis model of assessment of insulin resistance; TC, total cholesterol; LDLC, low-density lipoprotein cholesterol; HDLC, high-density lipoprotein cholesterol; TG, triglycerides; ALT, alanine transferase; AST, aspartate transferase; GGT, gamma-glutamyl transpeptidase; SBP, systolic blood pressure; DBP, diastolic blood pressure.

**Table S5. Between-group differences in the presence of the metabolic syndrome and its components according to per protocol analysis (n=127).**

|  | **MDG vs. SCG** | | | | **MLG vs. SCG** | | | | **MLG vs. MDG** | | | |
| --- | --- | --- | --- | --- | --- | --- | --- | --- | --- | --- | --- | --- |
|  | **RR** | **95%CI** | **P*** | **P**** | **RR** | **95%CI** | **P*** | **P**** | **RR** | **95%CI** | **P*** | **P**** |
| Increased WC ^a^ | 0.40 | 0.05, 3.08 | 0.3 | 0.4 | 0.13 | 0.02, 0.99 | 0.02 | 0.09 | 0.36 | 0.12, 1.08 | 0.1 | 0.2 |
| Increased TG ^b^ | 0.86 | 0.40, 1.87 | >0.9 | >0.9 | 0.66 | 0.30, 1.44 | 0.9 | >0.9 | 0.77 | 0.34, 1.71 | >0.9 | >0.9 |
| Decreased HDLC ^c^ | 0.76 | 0.40, 1.44 | 0.4 | 0.2 | 0.34 | 0.17, 0.66 | 0.001 | 0.001 | 0.44 | 0.24, 0.83 | 0.01 | 0.008 |
| Hypertension ^d^ | 0.90 | 0.48, 1.68 | 0.7 | 0.6 | 0.51 | 0.28, 0.92 | 0.06 | 0.9 | 0.56 | 0.31, 1.02 | 0.1 | 0.5 |
| Hyperglycemia ^e^ | 0.67 | 0.35, 1.28 | 0.5 | 0.7 | 0.64 | 0.33, 1.23 | 0.5 | 0.7 | 0.95 | 0.50, 1.81 | 0.9 | 0.8 |
| Presence of MS ^f^ | 0.77 | 0.41, 1.45 | 0.4 | 0.9 | 0.35 | 0.19, 0.65 | 0.001 | 0.09 | 0.46 | 0.24, 0.86 | 0.02 | 0.07 |

Results are presented as relative risk (95% confidence interval).

* P-value for the difference between groups at the end of the study, as derived from generalized linear models (binomial distribution with logit link function; Bonferroni correction was applied to adjust for multiple comparisons). All models were adjusted for participants’ age, sex, baseline levels of the dependent variables and continuous positive airway pressure use (h/week). Statistical significance level was set at 0.05. ** P-value after further adjustment for body-weight change (%).

^a^ >102 cm for males and >88 cm for females. ^b^ ≥1.7 mmol/L (≥150 mg/dL) or reception of lipid-lowering medication. ^c^ <1.0 mmol/L (<40 mg/dL) for males and <1.3 mmol/L (<50 mg/dL) for females, or reception of relevant medication. ^d^ systolic blood pressure ≥130 mm Hg or/and diastolic blood pressure ≥85 mm Hg, or reception of antihypertensive medication. ^e^ fasting glucose levels ≥5.6 mmol/L (≥100 mg/dL) or reception of antidiabetic medication. ^f^ According to the criteria proposed by Alberti et al.^[[3]](#footnote-3)^.

MDG, Mediterranean diet group; SCG, standard care group; MLG, Mediterranean lifestyle group; RR, relative risk; CI, confidence interval; WC, waist circumference; TG, triglycerides; HDLC, high-density lipoprotein cholesterol; MS, metabolic syndrome.

1. Alberti KG, Eckel RH, Grundy SM, et al. Harmonizing the metabolic syndrome: a joint interim statement of the International Diabetes Federation Task Force on Epidemiology and Prevention; National Heart, Lung, and Blood Institute; American Heart Association; World Heart Federation; International Atherosclerosis Society; and International Association for the Study of Obesity. Circulation 2009; 120: 1640-1645. [↑](#footnote-ref-1)
2. Alberti KG, Eckel RH, Grundy SM, et al. Harmonizing the metabolic syndrome: a joint interim statement of the International Diabetes Federation Task Force on Epidemiology and Prevention; National Heart, Lung, and Blood Institute; American Heart Association; World Heart Federation; International Atherosclerosis Society; and International Association for the Study of Obesity. Circulation 2009; 120: 1640-1645. [↑](#footnote-ref-2)
3. Alberti KG, Eckel RH, Grundy SM, et al. Harmonizing the metabolic syndrome: a joint interim statement of the International Diabetes Federation Task Force on Epidemiology and Prevention; National Heart, Lung, and Blood Institute; American Heart Association; World Heart Federation; International Atherosclerosis Society; and International Association for the Study of Obesity. Circulation 2009; 120: 1640-1645. [↑](#footnote-ref-3)
